# Two Novel *AGXT* Mutations Cause the Infantile Form of Primary Hyperoxaluria Type I in a Chinese Family: Research on Missed Mutation

**DOI:** 10.3389/fphar.2019.00085

**Published:** 2019-02-06

**Authors:** Xiulan Lu, Weijian Chen, Liping Li, Xinyuan Zhu, Caizhi Huang, Saijun Liu, Yongjia Yang, Yaowang Zhao

**Affiliations:** ^1^The Laboratory of Genetics and Metabolism, Hunan Children’s Research Institute (HCRI), Hunan Children’s Hospital, University of South China, Changsha, China; ^2^Pediatric Intensive Care Unit, Hunan Children’s Hospital, University of South China, Changsha, China; ^3^Department of Pathology, Hunan Children’s Hospital, University of South China, Changsha, China; ^4^BGI-China, Shenzhen, China; ^5^Department of Urinary Surgery, Hunan Children’s Hospital, University of South China, Changsha, China

**Keywords:** PH1 infantile type, AGXT, mutation, whole exome sequencing, nephrocalcinosis

## Abstract

Primary hyperoxaluria type 1 (PH1) is a rare metabolic disorder characterized by a defect in the liver-specific peroxisomal enzyme alanine-glyoxylate and serine-pyruvate aminotransferase (AGT). This disorder results in hyperoxaluria, recurrent urolithiasis, and nephrocalcinosis. Three forms of PH1 have been reported. Data on the infantile form of PH1 are currently limited in literature. Despite the fact that China is the most populated country in the world, only a few *AGXT* mutations have been reported in several Chinese PH1 patients. In the present study, we investigated a Chinese family in which two siblings are affected by the infantile form of PH1. Sanger sequencing was carried out on the proband, but the results were misleading. Two novel missense mutations (c.517T > C/p.Cys173Arg and c.667A > C/p.Ser223Arg) of the *AGXT* gene were successfully detected through whole-exome sequencing. These two mutations occurred in the highly conserved residues of the AGT. Four software programs predicted both mutations as the cause of the disease. A postmortem examination was performed and revealed the occurrence of global nephrocalcinosis on both kidneys. The crystals were collected and analyzed as calcium oxalate monohydrate. This study extends the knowledge on the clinical phenotype–genotype correlation of the *AGXT* mutation. That is, (i) two novel missense mutations were identified for the infantile form of PH1 and (ii) the same *AGXT* genotype caused the same infantile form of PH1 within the family.

## Introduction

Rare diseases burden societies and families (Jia and Shi, 2017). The natural history of a rare disease is often poorly characterized owing to insufficient knowledge, misdiagnoses, missed diagnoses, and incurability (Ni and Shi, 2017). Primary hyperoxaluria type 1 (PH1) is an inherited metabolic disorder caused by alanine-glyoxylate and serine-pyruvate aminotransferase (AGT) deficiency (Coulter-Mackie et al., 1993–2017). AGT is a hepatic peroxisomal enzyme that catalyzes the conversion of glyoxylate to glycine. When AGT activity is deficient, glyoxylate is converted to oxalate, which then forms insoluble calcium salt deposits in the kidney and other organs (Williams et al., 2009). Human PH1 can be classified into the following forms according to clinical severity: the infantile form with early renal insufficiency, the late-onset form with a good prognosis, and the most common form with recurrent urolithiasis or nephrocalcinosis that is usually accompanied by renal insufficiency (Jellouli et al., 2016). The infantile form is the most severe (Jellouli et al., 2016). The genetic basis for PH1 has been identified in at least 190 published *AGXT* gene (coding AGT) mutations (Coulter-Mackie et al., 1993–2017; Isiyel et al., 2016). However, data on the infantile form of PH1 are limited (less than 1/5 of PH1 patients have the infantile form) (Kurt-Sukur et al., 2015; Jellouli et al., 2016). Families with the infantile form of PH1 usually include only one affected patient (van Woerden et al., 2004; Kurt-Sukur et al., 2015; Jellouli et al., 2016; Cui et al., 2017; Soliman et al., 2017). This rate of occurrence makes the delineation of the clinical phenotype–genotype correlation of the *AGXT* mutation–infantile form of PH1 difficult. Meanwhile, China is the most populated country in the world, but cases of *AGXT* mutations in the Chinese population are rarely reported (Yuen et al., 2004; Li et al., 2014; Wang et al., 2016; Cui et al., 2017). The characteristics of the *AGXT* mutation spectrum in the Chinese population remain unclear. In this study, we investigated a Chinese family in which two siblings carried the c.517T > C/p.Cys173Arg and c.667A > C/p.Ser223Arg mutations of the *AGXT* gene. We discovered that the same *AGXT* genotype causes the same infantile form of PH1within a family.

## Results

### Clinical Data

#### Patient 1

**FIGURE 1:**
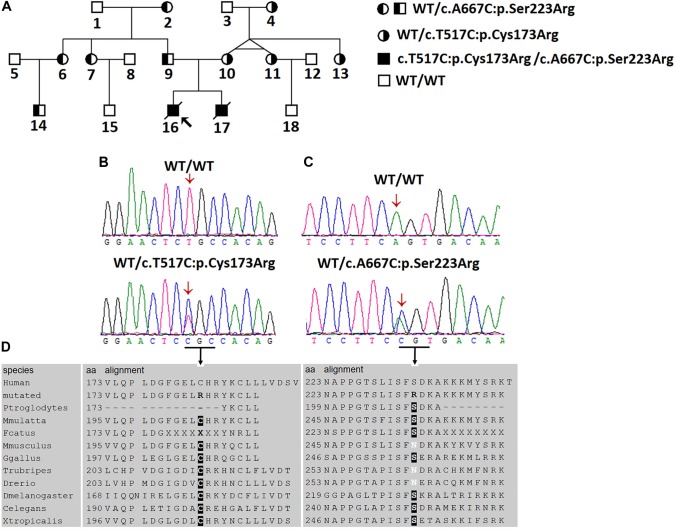
Two novel *AGXT* mutations in a Chinese family with the infantile form of primary hyperoxaluria type I (PH1). **(A)** Investigated Chinese family with PH1. Subjects 16 and 17 suffered from the infantile form PH1. Subjects 4, 10, 11, and 13 were affected by benign urolithiasis at least once, and other family members were asymptomatic. **(B,C)** Sanger sequencing revealed the *AGXT* gene mutations, c.517T > C/p.Cys173Arg and c.667A > C/p.Ser223Arg. **(D)** Both mutations occurred on the conserved residues of the AGT protein.

A boy, aged 4 months and 7 days (Subject 16, Figure 1A) and suffering from recurrent diarrhea (7–8 times per day) of unknown etiology, was referred to our hospital. The boy was born in Central China (Hunan Province, Han Chinese) and was the first child of non-consanguineous parents. His birth weight was 3050 g after full-term gestation without any medical problem. When the boy was admitted to our hospital, his rectal temperature was 36.5°C, blood pressure was 130/90 mmHg, pulse rate was 163 beats/min, and breathing rate was 8 breaths/min. He had severe hyponatremia, metabolic acidosis, and anemia (Table 1). His urine analysis results showed proteinuria (Supplementary Table S1), and his renal ultrasonography revealed that both his kidneys were small and exhibited mildly increased echogenicity. The patient progressed rapidly to end-stage renal disease (ESRD) at the age of 4 months and 12 days. The patient died at 4 months and 17 days.

**Table 1:** Biochemical features of two patients with infantile form of primary hyperoxaluria type 1.

		III:3	III:4	Normal range
Blood test + EPO	WBC (× 10^9^/L)	6.11	3.4	5–12
	N%	0.41	0.46	0.20–0.60
	L%	0.46	0.46	0.20–0.70
	RBC (× 10^12^/L)	2.68	2.12	3.5–5.5
	Hb (g/L)	58	55	110–160
	PLT (× 10^9^/L)	281	228	100–400
	EPO (mIU/mL)	1.85	1.76	2.59–18.50
Renal function	BUN (mmol/L)	23.64	27.24	1.8–8.2
+ Liver Function	Cr (μmol/L)	512.9	766	20–120
test + Myocardial	UA (μmol/L)	355.1	307	90–350
enzyme function	CK (IU/L)	774	380	38–174
	CK-MB (IU/L)	24	14	0–24
	LDH (IU/L)	358	185	0–450
	MB (IU/L)	487.92	560.2	0–90
	NT-ProBNP (pg/mL)	> 25000	25000	0–236
	TBIL (μmol/L)	3.5	2.71	3.40–17.0
	DBIL (μmol/L)	1.9	2.4	0–6.0
	IBIL (μmol/L)	1.6	0.31	3–17.0
	TP (g/L)	53.6	41.8	55–80
	ALB (g/L)	34.1	27.3	35–55
	GLO (g/L)	19.5	14.5	20–35
	ALT (IU/L)	38	49.6	0–40
	AST (IU/L)	40	28.6	0–40
Electrolyte analysis	K^+^ (mmol/L)	5.26	5.52	3.5–5.5
	Na^+^(mmol/L)	114.3	116.91	135–145
	Cl^-^ (mmol/L)	91.7	82.5	96–108
	Ca^2+^ (mmol/L)	2.64	1.43	2.1–2.7
	Mg^2+^ (mmol/L)	0.78	0.64	0.7–1.10
	P (mmol/L)	1.81	2.5	1.45–2.10
Arterial Blood	pH	7.26	7.28	7.35–7.45
gas + Lactic acid	HCO_3_^-^ (mmol/L)	9	10.8	22–27
	BE (mmol/L)	-16.6	-15.9	-3–3
	PCO_2_ (mmHg)	20	23	35–45
	PO_2_ (mmHg)	109	228	80–100
	Lac (mmol/L)	1.9	0.9	0.50–2.00


*Urine test results are presented in Supplementary Table S1. The red values in the table mean the results that are beyond the normal range.*

#### Patient 2

This child is the younger brother of patient 1 (Subject 17, Figure 1A). The boy also suffered from recurrent diarrhea (5–6 times per day) at the age of 4 months and 6 days. His birth weight was 3400 g at full-term gestation and presented no medical problem. His laboratory test results indicated abnormal liver and renal functions and anemia (Table 1). Urine analysis results showed proteinuria (Supplementary Table S1). ESRD developed at the age of 4 months and 15 days for the patient. Blood dialysis therapy was performed for 28 days. The patient died at the age of 5 months and 5 days. In brief, all the clinical data of the two patients were standardized and deposited into eRAM (Jia et al., 2018).

### First Round of Sanger Sequencing of the *AGXT* Gene

Given the clinical findings with unknown ESRD, we suspected the occurrence of a metabolic disease. After examining the proband affected by PH1 (June 2008), we synthesized the primers for all the exons and exon–intron boundaries of the *AGXT* gene (Supplementary Table S2). Sanger sequencing was routinely performed on the coding regions and the exon–intron boundaries of the *AGXT* gene (NM_000030) in subjects 16, 9, and 10. We detected a missense mutation on exon 4 (c.517T > C/p.Cys173Arg; Figure 1B) and validated the mutation by Sanger sequencing using reverse primer. The mutation originated from the patients’ healthy mother (Figure 1A). At this Cys173 amino acid position, the p.Cys173Tyr mutation associated with severely reduced catalytic activity was previously reported on a PH1 patient (Von Schnakenburg and Rumsby, 1998). Human PH1 is clearly caused by *AGXT* recessive mutation. However, no other pathogenic variant of the *AGXT* gene was detected in the trios-family despite the successful amplification and sequencing of all the exons and boundaries of *AGXT*. The PH1 diagnosis in the family was stalled because of another mutated *AGXT* allele, which was not detected in the family.

### Next-Generation Sequencing for Four Family Members

On December 2016, subject 17 was admitted in our hospital because of clinical symptoms similar to those in subject 16. The high phenotypic similarity of the two infants prompted us to conduct next-generation sequencing on the family (Figure 1A; whole-exome sequencing on subjects 9, 10, 16, and 17). We envisage that next generation sequencing may generate new data for the diagnosis of the disease in the family (Shen, 2018). We hypothesized that a recessive metabolic disorder affected the family. Considering non-consanguineous mating, we mainly focused on the compound heterozygous mutations in the family. Common variants in public databases (the variant was neither found in ExAC nor 1000 Genomes) and in-house databases were filtered out. Given that both patients died of ESRD, we focused on the genes involved in renal disorders (Supplementary Table S3). We detected the two heterozygous variants (chr2:241810859T > C and chr2:241813466A > C) of the *AGXT* gene segregated with PH1 in the family (Figure 1 and Supplementary Figure S1). Both *AGXT* variants were missense variants and caused the disease according to the reports of four prediction software programs (Table 2).

**Table 2:** Functional predictions of the two mutations of *AGXT* in this study.

Variant	Mutation taster^c^	Polyphen^d^	PROVEAN^e^	SIFT^f^
c.517T > C/p.Cys173Arg	Disease-causing prob: 0.999999999999603^a^	Probably damaging score: 1 (sensitivity: 0; specificity: 1)	Deleterious score: -10.79^b^	DAMAGING
c.667A > C / p.Ser223Arg	Disease-causing prob: 0.999994390365302	Probably damaging score: 0.999 (sensitivity: 0.14; specificity: 0.99)	Deleterious score: -4.26	DAMAGING


*^*a*^, The probability value is the probability of the prediction, i.e., a value close to one indicates a high “security” of the prediction; ^*b*^, PREDICTION (cutoff = -2.5); ^*c*^, http://www.mutationtaster.org/; ^*d*^, http://genetics.bwh.harvard.edu/pph2/; ^*e*^, http://provean.jcvi.org/genome_submit_2.php; ^*f*^, https://sift.bii.a-star.edu.sg/www/Extended_SIFT_chr_coords_submit.html.*

The variant chr2:241810859T > C was identified in exon 4 of the *AGXT* gene and harbored a change in residue 173 from cysteine to arginine (Figure 1B). The mutation of *AGXT*-exon 4 (chr2:241810859T > C) was detected as mentioned above. By contrast, variant chr2:241813466A > C was identified in exon 6 of the *AGXT* gene and harbored a change in residue 223 from serine to arginine. The *AGXT-*exon 6 mutation (chr2:241813466A > C) was missed in the first round of Sanger sequencing (mentioned above; detailed data are presented in the Discussion section). After re-synthesis, a new pair of primers (2AGXT6F: 5′-CATCTCCCCTGCTATCGTGTAC-3′; 2AGXT6R: 5′-CCTCAGTCCTTTCCTGGTCAC-3′, predicted PCR product size: 498 bp) was used for *AGXT*-exon 6, and the c.667A > C/p.Ser223Arg mutation (Figure 1C) was detected in the second round of Sanger sequencing.

### AGT-Ma and AGT-Mi Detection

The wild-type *AGXT* gene can carry two polymorphic variants: the rs4426527 (p.Ile340Met) and the less frequent minor haplotype (rs34116584, p.Pro11Leu). In this study, we utilized the results of next-generation sequencing (Supplementary Figure S1). However, we discovered that none of the family members carried any of the two variants. Therefore, both mutations are on the major haplotype.

### Postmortem and Nephrocalcinosis

**FIGURE 2:**
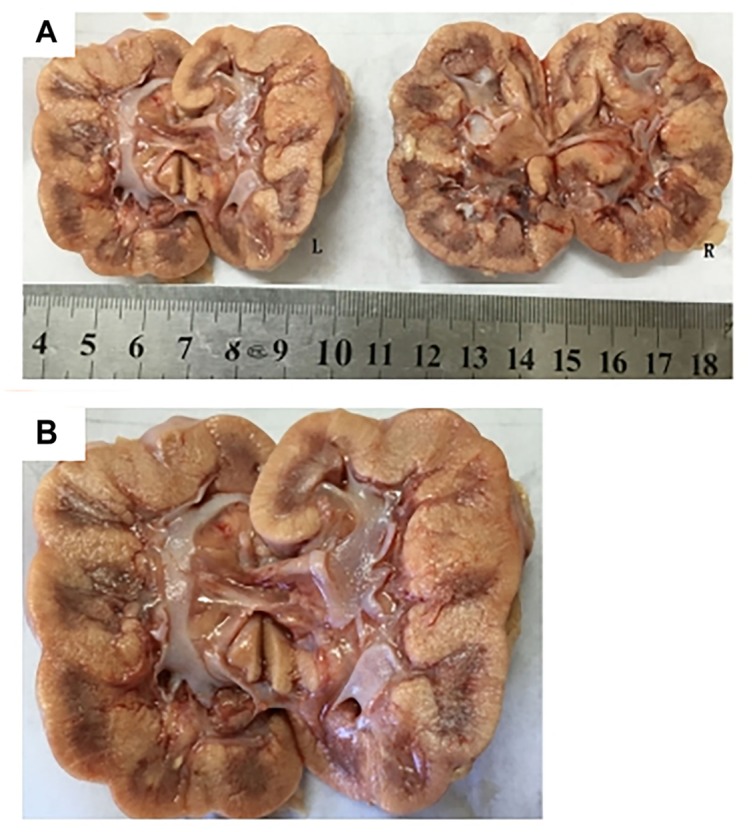
Postmortem examination was performed on III:4 of the family. **(A,B)** No visible stone was found on the bilateral kidneys of III:4.

**FIGURE 3:**
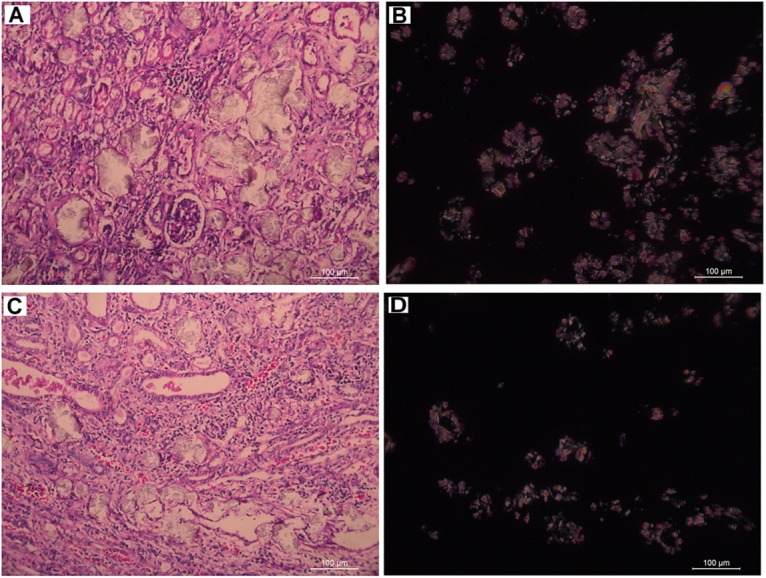
Renal tissue of III:4 viewed under the polariscope. **(A,B)** medullary nephrocalcinosis, **(C,D)** cortical nephrocalcinosis. **(A,C)** Under Single polarization **(B,D)** Under crossed nicols.

**FIGURE 4:**
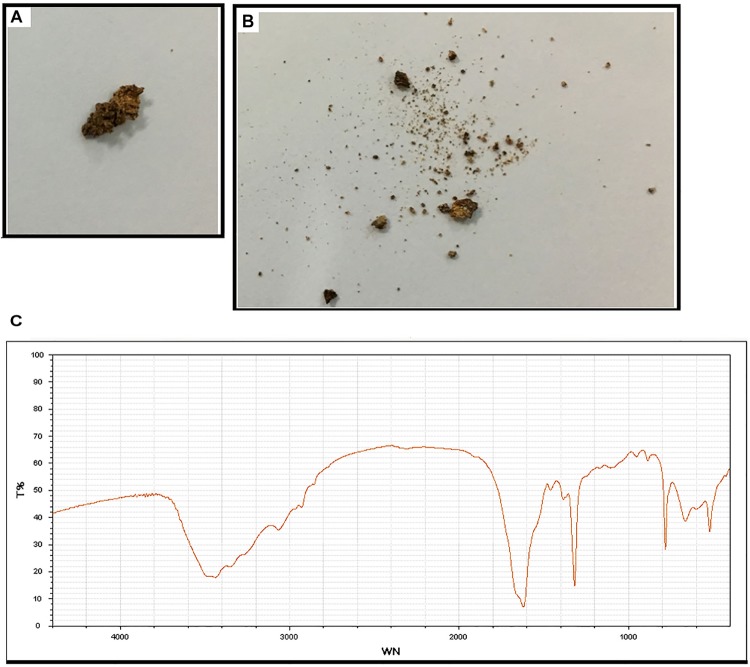
Composition analysis of the renal calcareous sediments. **(A,B)** Renal tissue (5 g) dried at high temperature (180°C) for 2 h. **(C)** Infrared spectroscopy of the sediments revealed the crystal as calcium oxalate monohydrate. An automatic infrared spectrum analysis system, LIIR-20 (approved by the Chinese FDA, No. 2008-2210004), was used in this study. T%, absorption frequency; WN,: wavenumber.

Subject 17 died of ESRD, and a postmortem examination was performed. No visible stone was observed inside both kidneys of the patient (Figure 2A,B). However, global nephrocalcinosis occurred in both kidneys, as observed under a polariscope (Figure 3). We obtained 5 g of the kidney tissue and placed the sample in a hot air oven for 2 h (Figure 4A,B). Crystals were collected for composition analysis (infrared spectroscopy test) and were identified as calcium oxalate monohydrate (Figure 4C).

## Discussion

Human AGT protein is a fold-type I pyridoxal 5′-phosphate (PLP)-dependent enzyme. The AGT molecule has a homodimeric structure of 2 × 43 kD/392 amino acids (Supplementary Figure S2; Williams et al., 2009). In this study, we identified two novel missense mutations (c.517T > C: p.Cys173Arg and c.667A > C:p.Ser223Arg) in the *AGXT* gene co-segregated with the infantile form of PH1 in a Chinese family. Both mutations, c.517T > C and c.667A > C, were not recorded in 500 in-house exome data and did not appear in 191 matched controls. Moreover, both mutations were absent in the Gnom_AD, ExAc, and 1000 Genomes public databases. Mutations on the amino acid position Cys173 of the AGT protein was reported previously (Von Schnakenburg and Rumsby, 1998; van Woerden et al., 2004). van Woerden et al. identified the p.Cys173Ter/ IVS1-1G *>* A compound heterozygous mutation on a 14-year-old patient with PH1 (van Woerden et al., 2004). The same position of p.Cys173Tyr mutation was also reported in a patient with PH1, as included in the HGMD public database^1^ (Von Schnakenburg and Rumsby, 1998).

In the crystal structure of AGT (Supplementary Figure S2), a change in Cys173 disrupts the alpha helix, which is on the exposed surface of the AGT protein; notably, Gly170, the most mutated residue on patients with PH1, is also on the alpha helix (Von Schnakenburg and Rumsby, 1998; Williams et al., 2009). We noted that amino acids 201–221 of the AGT protein constitute the consensus sequence of the PLP cofactor binding site (Robbiano et al., 2010; Oppici et al., 2013). Without direct contact with a cognate monomer (Supplementary Figure S2), the p.Ser223Arg mutation considered in the present study was near the 201–221 PLP cofactor binding site and probably disrupted AGT-PLP binding.

**FIGURE 5:**
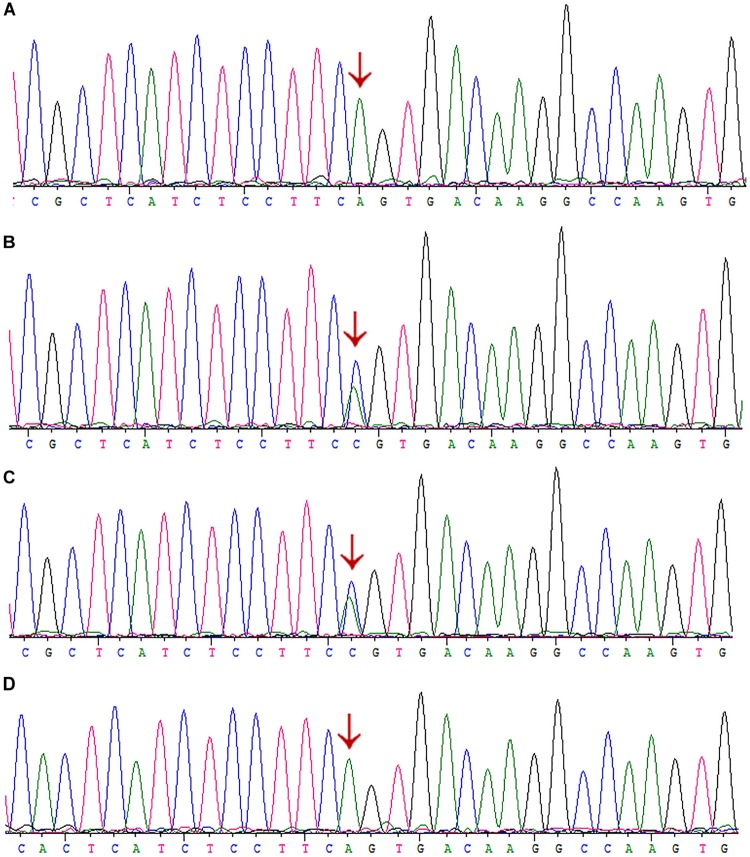
Sanger sequencing of AGXT-exon6 c.667A>C/S223R mutation by different primers. **(A)** Exon 6 of III:3 was PCR-amplified using the primer AGXT6F/AGXT6R and Sanger sequenced using the AGXT6F as the sequencing primer. **(B)** Exon 6 of III:3 was PCR-amplified using the primer 2AGXT6F/2AGXT6R and Sanger sequenced using the 2AGXT6F as the sequencing primer. **(C)** 2AGXT6F/2AGXT6R-amplified fragments Sanger-sequenced by AGXT6F. **(D)** AGXT6F/AGXT6R-amplified fragments Sanger-sequenced by 2AGXT6F.

Interestingly, we did not detect the exon 6 mutation of the *AGXT* gene (Figure 5A–D) in the family when AGXT6F/AGXT6R (for primer position, see Supplementary Figure S3) was used as the PCR primer. This finding is misleading for the diagnosis of PH1 in the family. However, the exon 6 mutation (c.667A > C/p.Ser223Arg) of the *AGXT* was detected through next-generation sequencing and was validated by the second round of Sanger sequencing (Figure 5B). The PCR and sequencing conditions for the second round of Sanger sequencing of *AGXT* exon 6 were as follows: the primers, 2AGXT6F/2AGXT6R (Supplementary Figure S3); predicted fragment length, 498 bp; and annealing temperature, 60°C. To determine why exon 6-c.667A > C was not detected in the first round of mutation screening, we sequenced the AGXT6F/AGXT6R-333 and 2AGXT6F/2AGXT6R-498 bp fragments by standard Sanger methods and used different sequencing primers. Accordingly, exon6-c.667A > C was not detected when the AGXT6R was used for PCR as the reverse primer (Table 3).

After careful analysis, we found that the AGXT6R primer was not located on SNPs (rs117619103 and rs78178548; Supplementary Figure S4), which were linked to the exon 6-c.667A > C mutation, but located between the SNPs, as confirmed by T-A cloning-Sanger sequencing (Supplementary Figure S5). Probably, in the presence of these SNPs, the genomic DNA formed a complex conformation (as predicted *in silico*, Supplementary Figure S6), and this condition may have disrupted the amplification of the disease allele in the PCR.

**FIGURE 6:**
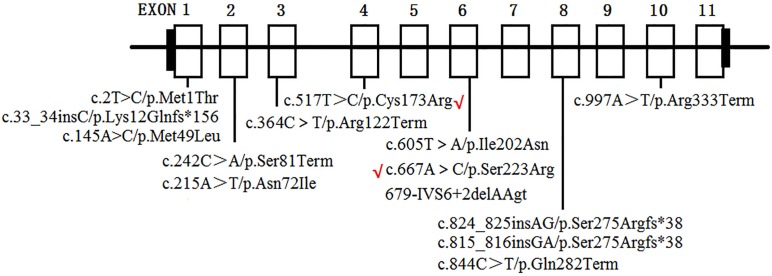
*AGXT* mutations on Chinese population. Novel mutations identified in the present study are labeled by red “√”.

To date, at least 190 *AGXT* mutations throughout the entire gene have been detected (Wang et al., 2016), and p.Gly170Arg, p.Phe152Ile, p.Ile244Thr, and c.33-34insC are four of the most common *AGXT* mutations (Williams and Rumsby, 2007; Fargue et al., 2013) in European and North American populations. This result suggests that *AGXT* mutations harbor “hot spots”. In East Asians, the p.Ser205Pro mutation is a PH1-specific mutation in Japanese patients (Kawai et al., 2012). Meanwhile, China, despite being the most populated country in the world, only have rare cases *AGXT* mutations in its population (Yuen et al., 2004; Li et al., 2014; Wang et al., 2016; Cui et al., 2017). To the best of our knowledge, only 11 *AGXT* gene mutations (Figure 6) on seven Chinese families have been reported in the country (Yuen et al., 2004; Li et al., 2014; Wang et al., 2016; Cui et al., 2017), and only one mutation carried the c.33-34insC hot spot *AGXT* mutation; the rest were not located on the hot spot (as mentioned above) nor on the Japanese p.Ser205 residue (Yuen et al., 2004; Li et al., 2014; Wang et al., 2016; Cui et al., 2017). Moreover, only two *AGXT* mutations, namely, c.2T > C/p.Met1? and c.824_825insAG/S275Rfs^∗^38, have been reported twice in the Chinese populations.

**Table 3:** Results of mutation screening for *AGXT* exon-6-mutation (c.667A > C/p.Ser223Arg) by different primers (Primers sequence and positions are provided in see Supplementary Figure S2).

PCR Amplification			Sanger sequencing	
	
primer-F	primer-R	Size	primer	exon6-c.667A > C
AGXT6F	AGXT6R	333bp	AGXT6F	(-)
AGXT6F	AGXT6R	333bp	AGXT6R	(-)
2AGXT6F	2AGXT6R	498bp	2AGXT6F	(+)
2AGXT6F	2AGXT6R	498bp	2AGXT6R	(+)
AGXT6F	AGXT6R	333bp	2AGXT6F	(-)
2AGXT6F	2AGXT6R	498bp	AGXT6F	(+)
2AGXT6F	2AGXT6R	498bp	AGXT6R	(+)


Thus, this situation is consistent with the two missense mutations (of the present study) being unreported elsewhere. All data indicated that *AGXT* mutation occurs in the Chinese population and may represent a new spectrum of *AGXT* mutations.

## Materials and Methods

### Ethics Statement

This study was approved by the ethics committee of the Hunan Children’s Hospital, Changsha City, China. The procedure of the committee conformed with the principles of the 2008 edition of the declaration of Helsinki.

Before this study, written informed consent was obtained from the guardian of the human subject for the publication of this study.

### Study Subjects

This study includes a Han, Chinese family (resides in the rural area of Hunan Province) with PH1 (Figure 1) and 496 ethnicity- and region-matched controls (the controls were selected from patients who came to our laboratory for GTG banding but who were excluded for having renal disorders).

In the family, subjects 16 and 17 were affected by the infantile form of PH1, whereas subjects 4, 10, 11, and 13 were affected by benign urolithiasis at least once in that a kidney stone composition analysis indicated that all the crystals were calcium oxalate monohydrate, and other family members were asymptomatic.

For each subject, genomic DNA was extracted from peripheral blood (2–5 mL in heparin sodium tubes) through the phenol/trichloromethane method, as described by the standard protocol.

### Sanger Sequencing

For mutation screening, the coding and intron-exon boundary regions of the *AGXT* gene (NM_000030, according to GRCh37/hg19) were amplified by PCR, using the primers synthesized by a local biotech company (BGI; Shenzhen, China) and designed with Primer3^2^. The sequencing reaction (BigDye 3.1 Kit, Applied Biosystems, Waltham, MA, United States) of the purified PCR products was performed according to the recommended procedure. The labeled PCR fragments were purified through 70% alcohol precipitation and electrophoresed on an ABI-A3500 genetic analyzer (Applied Biosystems, Waltham, MA, United States). The primer sequences, PCR conditions, and sequencing are shown in Supplementary Table S2.

For the validation of the AGXT-exon6 mutation (c.667A > C: p.Ser223Arg), two pairs of primers were used as presented in Supplementary Figure S3.

### Whole Exome Sequencing

The whole exome sequencing in the present study was performed according to the pipeline as reported elsewhere (Jin et al., 2018). In brief, two micrograms of genomic DNA for each sample (Subjects 16 and 17, Figure 1) was sheared into approximately 200-bp fragments. The fragments were enriched with ligation-mediated PCR. Exome capture (NimbleGen 2.1M HD array) was performed according to the manufacturer’s instructions (Roche NimbleGen, Inc., Madison, WI, United States). The captured library was sequenced on a HiSeq2000 sequencing platform (90 bp end reads, Illumina Inc., San Diego, CA, United States), and the Illumina base calling software V1.7 was used for the analysis of raw image files with default parameters. Clean reads were mapped to the reference human genome (hg19) ^3^, by using the BWA (Burrows–Wheeler Aligner) program with, at most, two mismatches ^4^. The alignment files (.bam) were generated with SAM tools ^5^ and reads of low mapping quality (< Q30) were filtered out. Clonal duplicated reads that may have been derived from PCR artifacts were removed with Picard Tools using default parameters^6^. Short read alignment and annotation visualization were performed by using the Integrative Genomics Viewer^7^. The percentage of alignment of the clean read to the exome regions was obtained by using our custom Perl scripts on the base of the alignment files. Single nucleotide variants (SNVs) and indels were detected with a genome analysis tool kit^8^. All detected SNVs and indels were comprehensively annotated by ANNOVAR^9^, including function implication (gene region, functional effect, mRNA GenBank accession number, amino acid change, cytoband, and so on) and allele frequency, in dbSNP, 1000 Genomes^10^, ESP6500 ^11^, and ExAc ^12^. Damaging missense mutations were predicted by SIFT ^13^, PolyPhen-2 ^14^, and MutationTaster ^15^.

## Author Contributions

YY and YZ designed the research. XL, WC, LL, XZ, CH, SL, YY, and YZ performed the sample collection and the research. YY and XL analyzed the experimental data and wrote the manuscript.

## Conflict of Interest Statement

The authors declare that the research was conducted in the absence of any commercial or financial relationships that could be construed as a potential conflict of interest.

## Footnotes

1 http://www.hgmd.cf.ac.uk

2 http://frodo.wi.mit.edu

3 genome.ucsc.edu

4 bio-bwa.sourceforge.net

5 samtools.sourceforge.net

6 broadinstitute.github.io/picard/

7 http://www.broadinstitute.org/igv/

8 www.broadinstitute.org/gatk

9 annovar.openbioinformatics.org/en/latest/

10 www.1000genomes.org

11 evs.gs.washington.edu/EVS/

12 exac.broadinstitute.org/

13 https://sift.bii.a-star.edu.sg/www/Extended_SIFT_chr_coords_submit.html

14 genetics.bwh.harvard.edu/pph2/

15 www.mutationtaster.org/
