# Supplementary material for: Two Novel AGXT Mutations Cause the Infantile Form of Primary Hyperoxaluria Type I in a Chinese Family: Research on Missed Mutation
Source: Front Pharmacol. 2019 Feb 6;10:85. doi: 10.3389/fphar.2019.00085 (PMC6372570; doi:10.3389/fphar.2019.00085)
Supplement: Supplementary file 1 [file Data_Sheet_1.PDF]

Fig. S1: Segregation analysis for *AGXT* gene variants in the family.

Note: [1] and [3] represents two intron variants; [2] represents c.667A>C/p.Ser223Arg and [4] represents c.517T>C/p.Cys173Arg

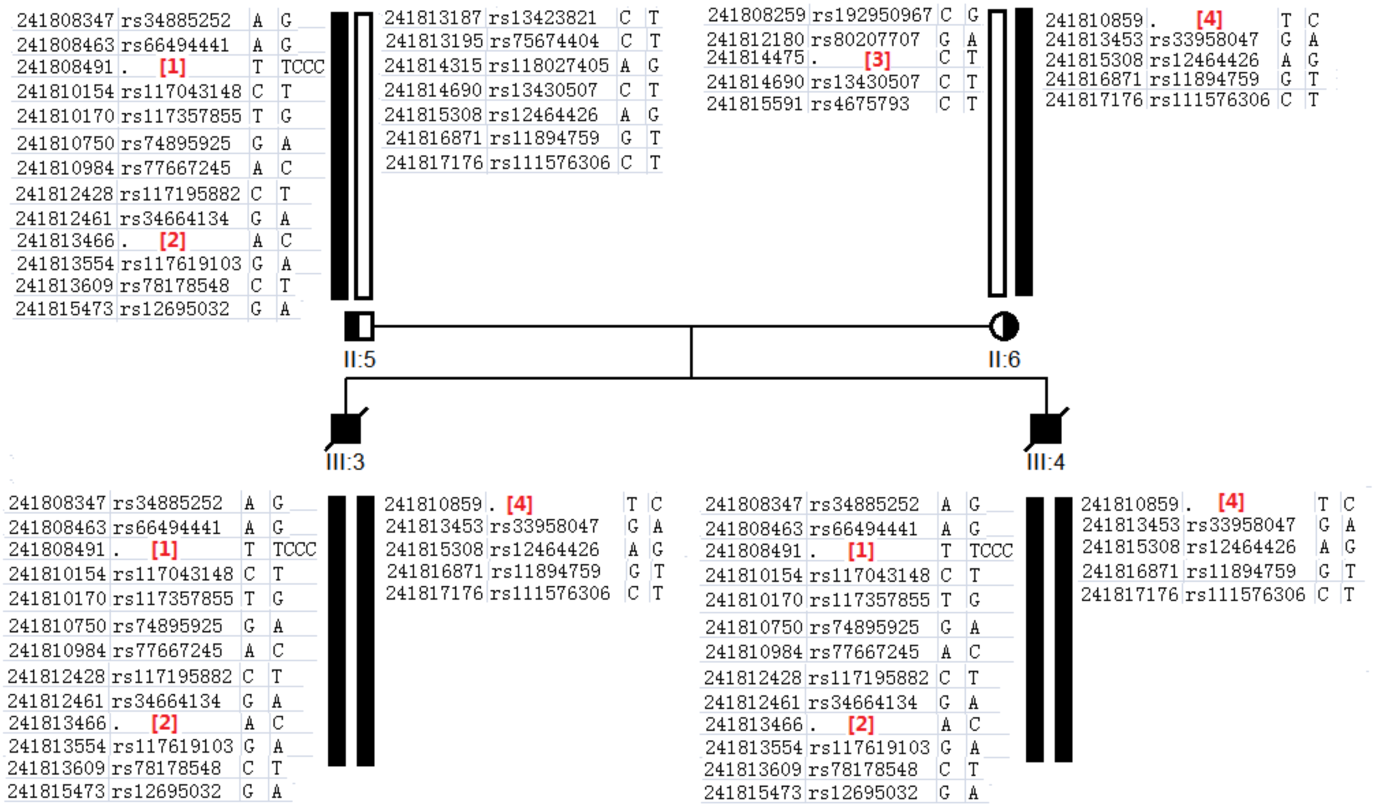

Fig. s2: Sites for p.Cys173 and p.Ser223 in the 3D structure (Cn3D-4.3.1) of Human AGT.

Note: AGT molecule has a homodimeric structure, one monomer blue and the other red. Both two residues are located on alpha helix without direct contact with the cognate monomer.

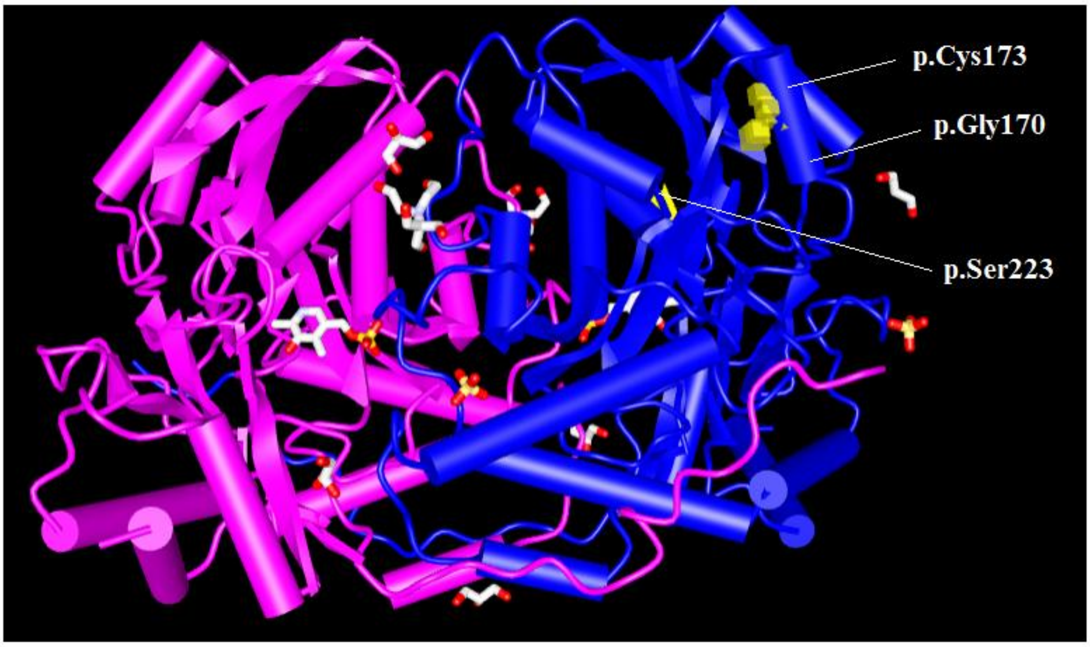

Fig. s3: Positions of *AGXT* exon 6 primers of this study. Two pairs of *AGXT* exon 6 primers were used, namely, AGXT6F/AGXT6R and 2AGXT6F/2AGXT6R.

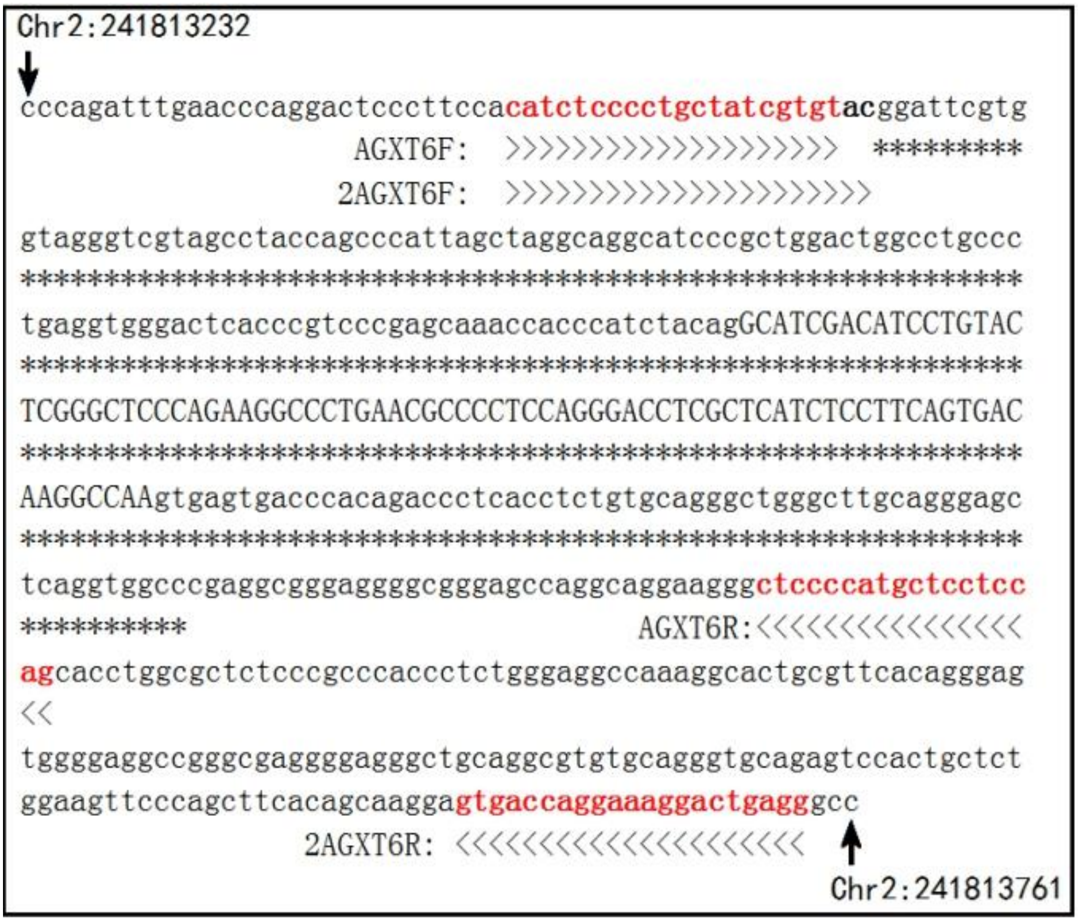

Fig. s4: AGXT6R primer position and near sequence characteristics.

Note: AGXT6R primer is between two nearing SNPs: rs117619103 and rs78178548.

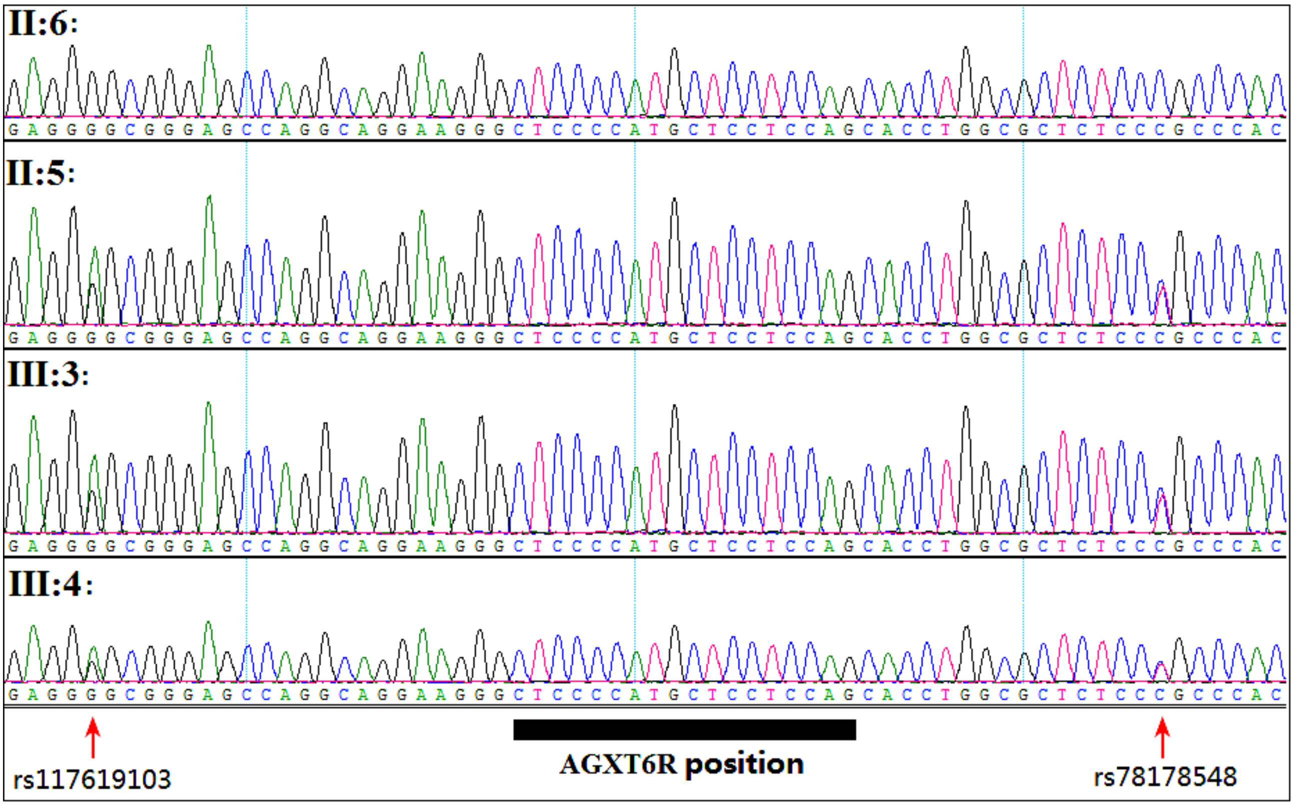

Fig. s5: T-A cloning of the PCR amplified 498-bp fragments (PCR primer 2AGXT6F/2AGXT6R) disclosed that two SNPs (rs117619103 and rs78178548) are linked to the exon 6-c.667A>C/p.Ser223Arg mutation in the family.

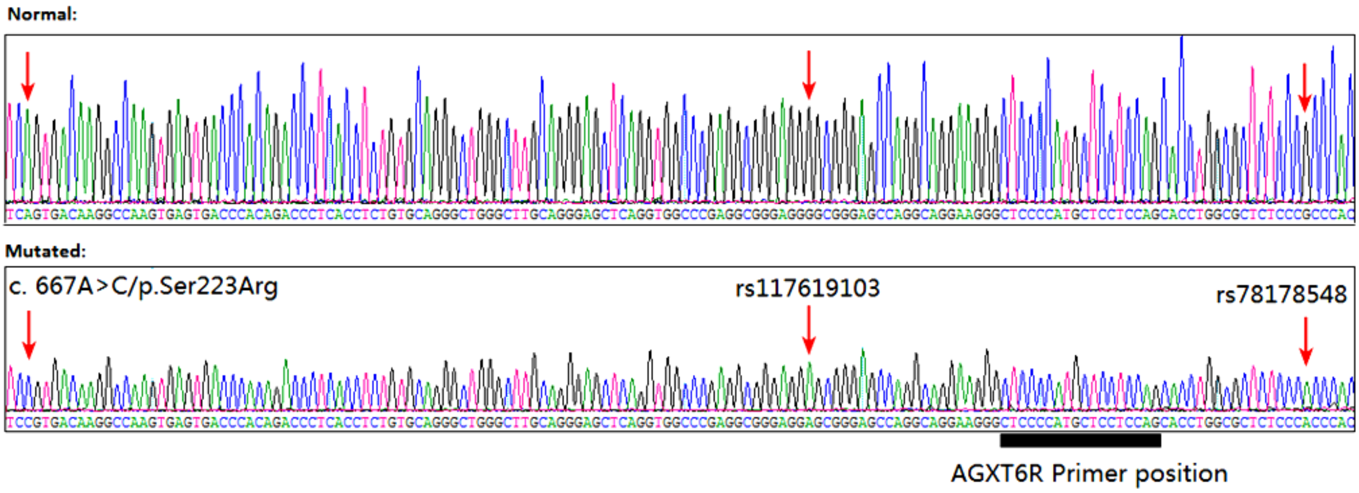

Fig. S6: DNA conformation analysis carried out by m-fold software. A: WT, only one large, loose-DNA hairpin structure formed; B: Mutated allele, exon 6-c.667A>C/p.Ser223Arg mutation linked to two SNPs (rs117619103 and rs78178548), two more stable, GC-rich DNA hairpin structure formed.

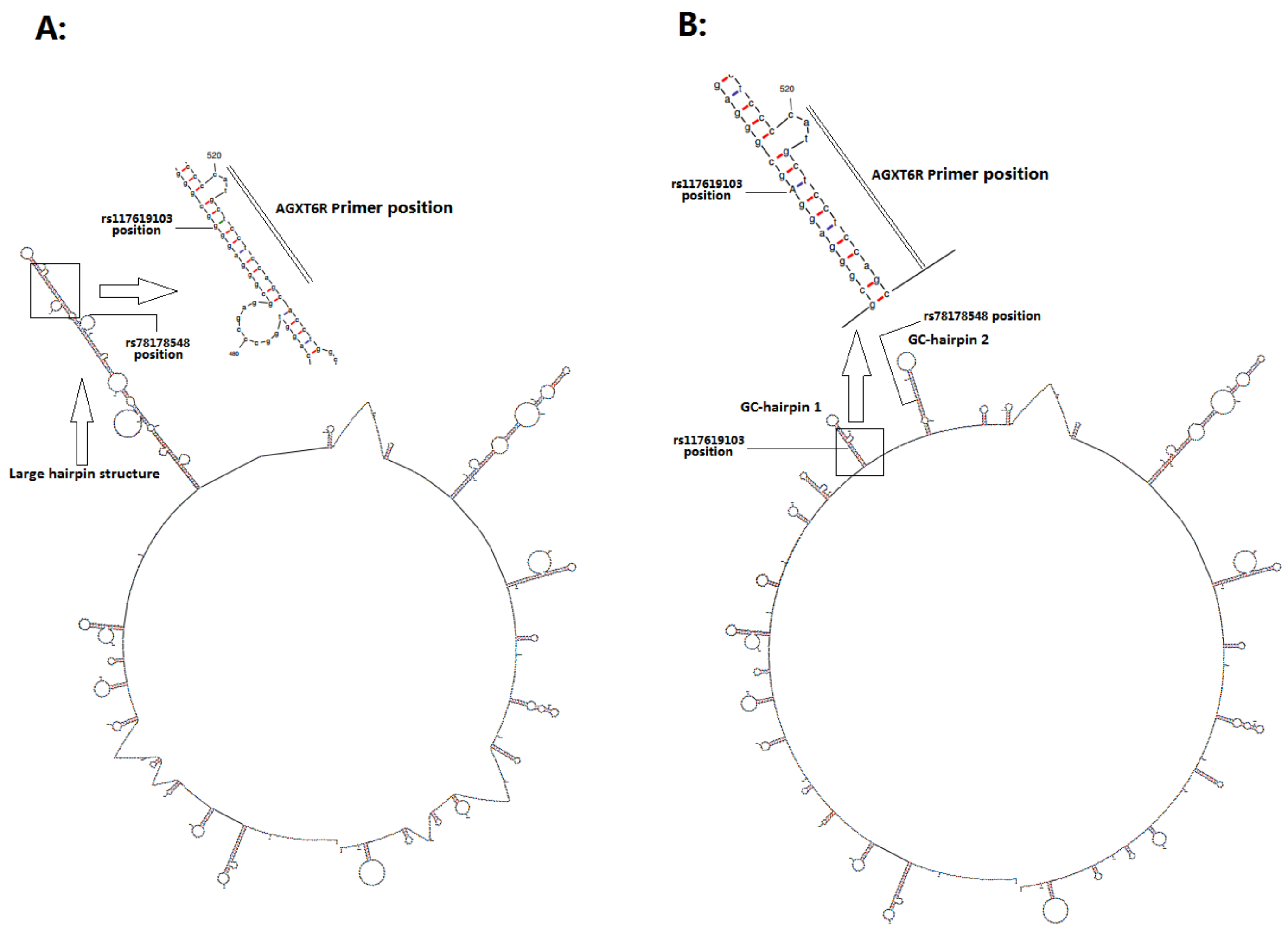

Table S1: Urine analysis results of two family members affected by renal damage.

Abbreviations	Projects	Results (III:3)	Results (III:4)	Reference range
Color	Color	yellow	yellow	Faint yellow～yellow
RBC UF	Red blood cell count	7.5/ul	5.6/ul	28d～1y:0.9～22.2/ul
WBC UF	White blood cell count	2.3/ul	1.4/ul	28d～1y:0.3～19.3/ul
EC	Epithelial cell	0.6/ul	1.3/ul	28d～1y:0.2～9.1/ul
CAST	Cast	0.1/ul	0.1/ul	<1y:0～0.66/ul
BACT	Bacteria	128.2/ul	11.6/ul	28d-1y:0～47.4/ul
SRC	Small round cell count	0.3/ul	1.0/ul	>28d:0～4.9/ul
YLC	Yeast-like cell count	0/ul	0/ul	>28d:0～0/ul
P.CAST	Pathologic cast count	0/ul	0/ul	>28d:0～0.26/ul
XTAL	Crystal count	0.2/ul	0.1/ul	0-10/ul
Large-RBC	Isomorphic red blood cell count	2.2/ul	2/ul	No reference range
Small-RBC	Dysmorphic red blood cell count	5.3/ul	3.6/ul	No reference range
Cond	Conductivity	12.4ms/cm	2ms/cm	28d-1y:0.3～23.1/ul
Glu	Glucose	+	+	negative
BIL	bilirubin	-	-	negative
KET	Ketone body	-	-	negative
SG	Specific gravity	1.005	1.005	Random urine:1.003-1.030
URO	Urobilinogen	-	-	negative
PH	Power of hydrogen	6.0	6.0	Morning urine:5.5-6.5
Pro	Protein	+	+	negative
NIT	Nitrite	-	-	negative

Table S2: Primers and polymerase chain reaction conditions for screening the *AGXT* mutation. Note: primers for the second round of exon6 amplification are presented in Figure S3.

**Exon**	**Forward (5'-3')**	**Reverse(5'-3')**	**Annealing temperature**	**Fragment Size**
**1-2**	ccatccaccaatcctcacct	ctgccagcttcaaacagagg	60℃	692bp
**3**	gagtccaccctcctcttcag	cagatgctaggatgggctga	60℃	391bp
**4**	atcagggaggagtagaggca	ctgcaccccataccatgtga	62℃	360bp
**5**	gacaggcaggagaaggcaa	aggagtgagtgtttccctgg	60℃	379bp
**6**	catctcccctgctatcgtgt	ctggaggagcatggggag	60℃	333bp
**7**	tggcccctgagcacaaat	gggctctagttggggttctt	60℃	374bp
**8**	tccaaagttctgaacccgga	cattgctgcatctgtccctg	58℃	440bp
**9**	accaaggcctgcagagtc	cttctcaggagccagtgtcc	62℃	296bp
**10**	tcctctggaacctgaagctg	ctgcaatctgggctttcgg	60℃	348bp

11	ggctgtcaactcccctcat	gactgcagctcatttggaa	60℃	488bp

Table S3: Variants that likely involved in renal disorders detected by Whole Exome Sequencing in the family.

III:4:

Filter	Inheritance	Gene Symbol	Function	MutationName	rsID	PVFD AF	Panel AlleleFreq
DUBIOUS	NN	CLCNKA	missense	NM_004070.3(CLCNKA): c.791C>A (p.Thr264Asn)	rs200145823	0.0044	0.0089
PASS	AR	MTR	coding-synon	NM_000254.2(MTR): c.3477C>T (p.(=))	rs117061132	0.0223	0.0446
PASS	AR	XDH	missense	NM_000379.3(XDH): c.406A>G (p.Met136Val)	.	0	.
PASS	AR	FASTKD2	missense	NM_001136194.1(FASTKD2): c.856G>A (p.Val286Ile)	.	0	.
PASS	AD	FN1	missense	NM_212482.1(FN1): c.3307A>C (p.Ile1103Leu)	rs13306364	0.0158	0.0268
PASS	AD	FN1	missense	NM_212482.1(FN1): c.2044A>G (p.Ile682Val)	.	0	.
PASS	AR	COL4A4	coding-synon	NM_000092.4(COL4A4): c.102A>G (p.(=))	rs3817617	0.0073	0.0312
PASS	AR	AGXT	missense	NM_000030.2(AGXT): c.517T>C (p.Cys173Arg)	.	0	.
PASS	AR	AGXT	missense	NM_000030.2(AGXT): c.667A>C (p.Ser223Arg)	.	0	.
DUBIOUS	AR	FANCD2	splice-5	NM_033084.3(FANCD2): c.1278+1delG	.	0	0.0045
PASS	AR	LAMB2	missense	NM_002292.3(LAMB2): c.2095G>C (p.Gly699Arg)	rs28364667	0.0043	.
PASS	NN	ROBO2	missense	NM_001128929.2(ROBO2): c.19C>A (p.Arg7Ser)	rs12171318	.	.
DUBIOUS	NN	ROBO2	missense	NM_001128929.2(ROBO2): c.73G>A (p.Val25Met)	rs2971727	.	.
PASS	AR	MUT	missense	NM_000255.3(MUT): c.1663G>A (p.Ala555Thr)	.	0.0005	.
PASS	AR	PKHD1	nonsense	NM_138694.3(PKHD1): c.11740C>T (p.Arg3914*)	.	0	.
PASS	AR	PKHD1	missense	NM_138694.3(PKHD1): c.3953A>T (p.His1318Leu)	rs200733734	0.0005	.
DUBIOUS	AD	SEC63	frameshift	NM_007214.4(SEC63): c.1605dupA (p.Pro536Thrfs*24)	.	.	.
PASS	AR	FAM20C	frameshift	NM_020223.3(FAM20C): c.957_958insT... (p.Ile320*)	.	.	.
PASS	AR	ATP6V0A4	missense	NM_020632.2(ATP6V0A4): c.1888G>A (p.Ala630Thr)	rs73730479	0.0376	0.0134

PASS	AD;AR	ROR2	missense	NM_004560.3(ROR2): c.2083G>A (p.Gly695Arg)	rs34431454	0.0302	0.0357
PASS	AR	INVS	missense	NM_014425.3(INVS): c.3141G>T (p.Lys1047Asn)	.	0	.
PASS	AR	SMPD1	missense	NM_000543.4(SMPD1): c.1598C>T (p.Pro533Leu)	rs199915216	0.0034	0.0089
DUBIOUS	AD;AR	WNK1	frameshift	NM_001184985.1(WNK1): c.1748dupA (p.Gln584Alafs*27)	.	.	.
DUBIOUS	AD;AR	VDR	intron	NM_001017536.1(VDR): c.148+58T>G	.	.	.
DUBIOUS	AD;AR	VDR	intron	NM_001017536.1(VDR): c.148+53C>G	.	.	.
PASS	AD	INF2	missense	NM_022489.3(INF2): c.1049C>T (p.Pro350Leu)	rs146529868	0.0092	0.0312
DUBIOUS	AD	INF2	frameshift	NM_022489.3(INF2): c.1337_1338delTG (p.Leu446Profs*6)	.	.	.
DUBIOUS	AD	INF2	frameshift	NM_022489.3(INF2): c.1339C[8] (p.Ser448Profs*112)	.	.	.
PASS	AD	TSC2	coding-synon	NM_000548.3(TSC2): c.1593C>T (p.(=))	rs45517180	0.0041	0.0179
PASS	AD	PKD1	missense	NM_001009944.2(PKD1): c.4051C>T (p.Arg1351Trp)	rs55840049	0.0024	.
PASS	AD	PKD1	missense	NM_001009944.2(PKD1): c.3931G>A (p.Ala1311Thr)	rs146169133	0.0048	0.0223
PASS	AD;AR	SCNN1B	intron	NM_000336.2(SCNN1B): c.1045-114T>C	.	.	0.014
PASS	AD;AR	ZNF423	missense	NM_015069.2(ZNF423): c.1537G>A (p.Gly513Ser)	rs201929999	0.0024	.
PASS	AR	MKS1	missense	NM_017777.3(MKS1): c.1543C>T (p.Arg515Cys)	.	.	.
DUBIOUS	AD	MYH9	intron	NM_002473.4(MYH9): c.333+50T>G	.	.	0.0089
PASS	AR	XPNPEP3	intron	NM_022098.3(XPNPEP3): c.182-165A>T	rs142797760	0.0018	0.0089
PASS	XD;XL	COL4A5	missense	NM_033380.2(COL4A5): c.2858G>T (p.Gly953Val)	rs78972735	0.0113	0
**III:3**							
DUBIOUS	NN	CLCNKA	missense	NM_004070.3(CLCNKA): c.860C>T (p.Ala287Val)	rs200268763	0.0333	0.0312
PASS	AR	BSND	missense	NM_057176.2(BSND): c.893G>A (p.Gly298Glu)	rs180858237	0.0036	.
PASS	AD	NOTCH2	intron	NM_024408.3(NOTCH2): c.751+289G>A	rs2124109	.	.
PASS	AR	MTR	coding-synon	NM_000254.2(MTR): c.3477C>T (p.(=))	rs117061132	0.0223	0.0446
PASS	AR	FASTKD2	missense	NM_001136194.1(FASTKD2): c.856G>A (p.Val286Ile)	rs758110363	0	.

PASS	AD	FN1	missense	NM_212482.1(FN1): c.2044A>G (p.Ile682Val)	.	0	.
PASS	AR	COL4A4	coding-synon	NM_000092.4(COL4A4): c.102A>G (p.(=))	rs3817617	0.0073	0.0312
PASS	AR	AGXT	missense	NM_000030.2(AGXT): c.517T>C (p.Cys173Arg)	.	0	.
PASS	AR	AGXT	missense	NM_000030.2(AGXT): c.667A>C (p.Ser223Arg)	.	0	.
PASS	NN	ROBO2	missense	NM_001128929.2(ROBO2): c.19C>A (p.Arg7Ser)	rs12171318	.	.
PASS	NN	ROBO2	missense	NM_001128929.2(ROBO2): c.73G>A (p.Val25Met)	rs78834776	.	.
PASS	NN	ROBO2	missense	NM_001128929.2(ROBO2): c.1787A>G (p.Tyr596Cys)	rs149389279	0.0018	0.0134
PASS	AR	PKHD1	nonsense	NM_138694.3(PKHD1): c.11740C>T (p.Arg3914*)	rs761704401	0	.
PASS	AR	ATP6V0A4	missense	NM_020632.2(ATP6V0A4): c.1888G>A (p.Ala630Thr)	rs73730479	0.0376	0.0134
PASS	AR	ESCO2	missense	NM_001017420.2(ESCO2): c.1522A>G (p.Ile508Val)	rs114956994	0.023	0.0179
DUBIOUS	AR	FANCC	missense	NM_000136.2(FANCC): c.1345G>A (p.Val449Met)	rs1800367	0.0101	0.0045
PASS	AD	MLH3	missense	NM_001040108.1(MLH3): c.3488G>A (p.Gly1163Asp)	rs28757011	0.0113	0.0312
PASS	AD	TSC2	coding-synon	NM_000548.3(TSC2): c.1593C>T (p.(=))	rs45517180	0.0041	0.0179
PASS	AD	PKD1	missense	NM_001009944.2(PKD1): c.4051C>T (p.Arg1351Trp)	rs55840049	0.0024	.
PASS	AD	PKD1	missense	NM_001009944.2(PKD1): c.3931G>A (p.Ala1311Thr)	rs146169133	0.0048	0.0223
PASS	AD;AR	SCNN1B	intron	NM_000336.2(SCNN1B): c.1045-114T>C	rs200146402	.	0.014
PASS	AR	ENO3	intron	NM_001976.4(ENO3): c.866-125C>T	rs2309347	.	0.0387
PASS	AD;AR	SLC4A1	missense	NM_000342.3(SLC4A1): c.2561C>T (p.Pro854Leu)	rs2285644	0.0175	0.0312
PASS	AR	MKS1	missense	NM_017777.3(MKS1): c.1543C>T (p.Arg515Cys)	rs775558298	.	.
PASS	AR	RAD51C	missense	NM_058216.1(RAD51C): c.71G>T (p.Arg24Leu)	.	0	0.0045
PASS	XD;XL	COL4A5	missense	NM_033380.2(COL4A5): c.2858G>T (p.Gly953Val)	rs78972735	0.0113	0
**II:6**							
PASS	AD	NOTCH2	intron	NM_024408.3(NOTCH2): c.751+289G>A	rs2124109	.	.
PASS	AR	MTR	utr-3	NM_000254.2(MTR): c.*2538A>G	rs148021206	0.0046	0.0089
PASS	AD	FN1	missense	NM_212482.1(FN1): c.3307A>C	rs13306364	0.015	0.0268

				(p.Ile1103Leu)		8	
PASS	AR	AGXT	missense	NM_000030.2(AGXT): c.517T>C (p.Cys173Arg)	.	0	.
PASS	AR	FANCD2	splice-5	NM_033084.3(FANCD2): c.1278+1delG	rs750338758	0	0.0045
PASS	NN	ROBO2	missense	NM_001128929.2(ROBO2): c.19C>A (p.Arg7Ser)	rs12171318	.	.
PASS	NN	ROBO2	missense	NM_001128929.2(ROBO2): c.73G>A (p.Val25Met)	rs78834776	.	.
PASS	NN	ROBO2	missense	NM_001128929.2(ROBO2): c.1787A>G (p.Tyr596Cys)	rs149389279	0.0018	0.0134
DUBIOUS	AR	NPHP3	utr-5	NM_153240.4(NPHP3): c.-77_-50delT…	rs781381515	.	.
PASS	AR	ERCC8	intron	NM_000082.3(ERCC8): c.1123-3_1123-2insT	rs777444521	.	0.0045
PASS	AR	PKHD1	nonsense	NM_138694.3(PKHD1): c.11740C>T (p.Arg3914*)	rs761704401	0	.
PASS	AR	PKHD1	missense	NM_138694.3(PKHD1): c.1835A>G (p.His612Arg)	rs369932370	0.001	.
PASS	AR	FAM20C	frameshift	NM_020223.3(FAM20C): c.957_958insT… (p.Ile320*)	rs774848096	.	.
PASS	AR	ATP6V0A4	missense	NM_020632.2(ATP6V0A4): c.1888G>A (p.Ala630Thr)	rs73730479	0.0376	0.0134
PASS	NN	PTPRE	intron	NM_006504.4(PTPRE): c.724-58A>G	rs2298198	0.0348	.
PASS	AR	SMPD1	missense	NM_000543.4(SMPD1): c.1598C>T (p.Pro533Leu)	rs199915216	0.0034	0.0089
PASS	AR	FAN1	coding-synon	NM_014967.4(FAN1): c.2403C>T (p.(=))	rs148908472	0.0036	0.0089
PASS	AD	TSC2	coding-synon	NM_000548.3(TSC2): c.1593C>T (p.(=))	rs45517180	0.0041	0.0179
PASS	AD	PKD1	missense	NM_001009944.2(PKD1): c.3931G>A (p.Ala1311Thr)	rs146169133	0.0048	0.0223
PASS	AD;AR	ZNF423	missense	NM_015069.2(ZNF423): c.1537G>A (p.Gly513Ser)	rs201929999	0.0024	.
PASS	AR	ENO3	intron	NM_001976.4(ENO3): c.866-125C>T	rs2309347	.	0.0387
PASS	AD;AR	SLC4A1	missense	NM_000342.3(SLC4A1): c.2561C>T (p.Pro854Leu)	rs2285644	0.0175	0.0312
PASS	AR	RAD51C	missense	NM_058216.1(RAD51C): c.71G>T (p.Arg24Leu)	.	0	0.0045
PASS	AD	PROKR2	missense	NM_144773.2(PROKR2): c.151G>A (p.Ala51Thr)	rs144994507	0.0096	0.0089
PASS	AD	PROKR2	missense	NM_144773.2(PROKR2): c.71T>G (p.Leu24Arg)	.	0	.
DUBIOUS	AR	XPNPEP3	intron	NM_022098.3(XPNPEP3): c.182-165A>T	rs142797760	0.0018	0.0089
PASS	XD;XL	COL4A5	missense	NM_033380.2(COL4A5):	rs78972735	0.011	0

				c.2858G>T (p.Gly953Val)		3	
**II:5:**							
PASS	AR	BSND	missense	NM_057176.2(BSND): c.893G>A (p.Gly298Glu)	rs180858237	0.0036	.
PASS	AD	NOTCH2	frameshift	NM_024408.3(NOTCH2): c.15C[2] (p.Pro6Argfs*27)	rs372504208	.	.
DUBIOUS	AD	NOTCH2	missense	NM_024408.3(NOTCH2): c.8C>T (p.Ala3Val)	rs200646249	0.0017	0.0134
DUBIOUS	AD	NOTCH2	missense	NM_024408.3(NOTCH2): c.7G>T (p.Ala3Ser)	rs782113557	0.0011	0.0134
PASS	AR	MTR	coding-synon	NM_000254.2(MTR): c.3477C>T (p.(=))	rs117061132	0.0223	0.0446
PASS	AR	XDH	missense	NM_000379.3(XDH): c.406A>G (p.Met136Val)	rs749589713	0	.
PASS	AR	FASTKD2	missense	NM_001136194.1(FASTKD2): c.856G>A (p.Val286Ile)	rs758110363	0	.
PASS	AD	FN1	missense	NM_212482.1(FN1): c.2044A>G (p.Ile682Val)	.	0	.
PASS	AR	COL4A4	coding-synon	NM_000092.4(COL4A4): c.102A>G (p.(=))	rs3817617	0.0073	0.0312
PASS	AR	AGXT	missense	NM_000030.2(AGXT): c.667A>C (p.Ser223Arg)	.	0	.
PASS	AR	LAMB2	missense	NM_002292.3(LAMB2): c.2095G>C (p.Gly699Arg)	rs28364667	0.0043	.
PASS	NN	ROBO2	missense	NM_001128929.2(ROBO2): c.19C>A (p.Arg7Ser)	rs12171318	.	.
PASS	NN	ROBO2	missense	NM_001128929.2(ROBO2): c.73G>A (p.Val25Met)	rs78834776	.	.
PASS	NN	ROBO2	missense	NM_001128929.2(ROBO2): c.1787A>G (p.Tyr596Cys)	rs149389279	0.0018	0.0134
PASS	AR	WDR19	missense	NM_025132.3(WDR19): c.1535G>A (p.Arg512Gln)	rs537283735	.	.
PASS	AR	MUT	missense	NM_000255.3(MUT): c.1663G>A (p.Ala555Thr)	rs753564352	0.0005	.
PASS	AR	PKHD1	missense	NM_138694.3(PKHD1): c.3953A>T (p.His1318Leu)	rs200733734	0.0005	.
PASS	AR	FAM20C	frameshift	NM_020223.3(FAM20C): c.957_958insT... (p.Ile320*)	rs774848096	.	.
PASS	AR	PGAM2	missense	NM_000290.3(PGAM2): c.478A>G (p.Ile160Val)	.	0	.
PASS	AR	ATP6V0A4	missense	NM_020632.2(ATP6V0A4): c.1888G>A (p.Ala630Thr)	rs73730479	0.0376	0.0134
PASS	AR	ESCO2	missense	NM_001017420.2(ESCO2): c.1522A>G (p.Ile508Val)	rs114956994	0.023	0.0179
PASS	AD;AR	ROR2	missense	NM_004560.3(ROR2): c.2083G>A (p.Gly695Arg)	rs34431454	0.0302	0.0357
PASS	AR	FANCC	missense	NM_000136.2(FANCC): c.1345G>A (p.Val449Met)	rs1800367	0.0101	0.0045

PASS	AR	INVS	missense	NM_014425.3(INVS): c.3141G>T (p.Lys1047Asn)	.	0	.
PASS	AR	DYNC2H1	intron	NM_001080463.1(DYNC2H1): c.3574-48A>T	rs79122459	0.0119	0.0491
PASS	AD;AR	WNK1	missense	NM_001184985.1(WNK1): c.446C>T (p.Ala149Val)	rs34880640	0.0129	0.0268
PASS	AD	MLH3	missense	NM_001040108.1(MLH3): c.3488G>A (p.Gly1163Asp)	rs28757011	0.0113	0.0312
PASS	AD	INF2	missense	NM_022489.3(INF2): c.1049C>T (p.Pro350Leu)	rs146529868	0.0092	0.0312
DUBIOUS	AD	PKD1	missense	NM_001009944.2(PKD1): c.4051C>T (p.Arg1351Trp)	rs55840049	0.0024	.
PASS	AD;AR	SCNN1B	intron	NM_000336.2(SCNN1B): c.1045-114T>C	rs200146402	.	0.014
PASS	AR	PHKG2	coding-synon	NM_000294.2(PHKG2): c.174A>T (p.(=))	rs56207641	0.0055	0.0089
PASS	AD;AR	SLC4A1	missense	NM_000342.3(SLC4A1): c.1151G>A (p.Arg384His)	rs13306776	0.0012	.
PASS	AR	MKS1	missense	NM_017777.3(MKS1): c.1543C>T (p.Arg515Cys)	rs775558298	.	.
PASS	AD;AR	PRODH	coding-synon	NM_016335.4(PRODH): c.1440C>T (p.(=))	rs5992333	0.0364	0.0312
